# IFNα induces CCR5 in CD4^+^ T-cells, causing its anti- HIV inefficiency and its subsequent pathogenic elevation, partially controlled by anti-HIV therapy

**DOI:** 10.21203/rs.3.rs-2813616/v1

**Published:** 2023-05-11

**Authors:** Hélène Le Buanec, Valérie Schiavon, Marine Merandet, Alexandre How-Kit, Hongshuo Song, David Bergerat, Céline Fombellida-Lopez, Armand Bensussan, Jean-David Bouaziz, Arsène Burny, Gilles Darcis, Mohammad M. Sajadi, Shyamasundaran Kottilil, Daniel Zagury, Robert C. Gallo

**Affiliations:** 1 Université de Paris; INSERM U976, HIPI Unit, Institut de Recherche Saint-Louis, F-75010 Paris, France.; 2 Laboratory for Genomics Foundation Jean Dausset-CEPH; Paris France; 3 Institute of Human Virology, School of Medicine, University of Maryland; Baltimore MD, 21201, USA, Department of Medicine, School of Medicine, University of Maryland, Baltimore, MD, 21201, USA.; 4 Laboratory of Infectious Diseases, GIGA-I3, GIGA-Institute University of Liege; 4000 Liege, Belgium; 5 Dermatology Department, Hôpital Saint-Louis, Assistance Publique-Hôpitaux de Paris (AP-HP), Paris, France; 6 Laboratory of Molecular Biology, Gembloux Agrobiotech,University of Liège ;Belgium; 7 Global Virus Network, Baltimore, MD 21201, USA.; 8 University of Maryland School of Medicine; Baltimore, MD 21201, USA, Program in Oncology, Marlene and Stewart Greenebaum Comprehensive Cancer Center, University of Maryland, Baltimore, MD 21201, USA.; 9 21CBIO ; Paris France.

## Abstract

Like EC, we find that ART-treated patients control serum IFNα concentration and show few immune cell alterations enabling a healthy but fragile medical status. However, treatment interruption leads to elevated IFNα reflecting virus production indicating that like EC, ART does not achieve a virological cure. The immune system becomes overwhelmed by multiple immune cell abnormalities as found in untreated patients. These are chiefly mediated by elevated IFNα inducing signaling checkpoints abnormalities, including PD1, in cytotoxic immune cells. Importantly, during acute infection, elevated IFNα correlated with HIV load and we found that IFNα enhances CCR5, the HIV coreceptor in CD4^+^ T-cells, impairing its anti-viral response and accounting for the pathogenic vicious cycle: HIV → IFNα ↗ → infected CD4^+^ T-cells ↗ →HIV ↗. This study opens immunotherapeutic perspectives showing the need to control IFNα in order to convert ART patients into EC.

## Introduction

Therapy for HIV infection was based on targeting specific steps in HIV replication and led to major clinical improvement and prognosis. However, there remain some medical issues (1,2,3,4,5,6) and the problem of residual HIV reservoirs (7,8), which lead to loss of virus control when therapy is interrupted (9,10). The chief clinical goal today is an attempt to reach a functional cure in which no further therapy is needed mimicking the elite controller (EC) status (0.5% of HIV-infected persons requiring no therapy for years or even decades) (11,12). To reach this end and in agreement with prior suggestions (13), we think a deeper understanding of pathogenesis may provide new insights for this purpose. In this context, we examined all blood immune cell types at different stages of an HIV immune reaction, comparing untreated HIV-infected EC with untreated infected non-EC (14). We found all immune cells to be altered in non-EC, leading us to suggest that in addition to HIV, which directly targets only a few immune cell types, the involvement of one or more mediators. We identified the main mediator as elevated circulating pathogenic IFNα, which is active in the earliest days of infection but avoided by ECs (14), possibly by their infection by a lower inoculum.

In this companion report, we further pursue these studies with a detailed analysis of all blood immune cells of HIV-infected non-EC that have received anti-retroviral therapy (ART) and compared these results with untreated patients. If therapy is interrupted in treated patients, HIV viremia returns demonstrating that anti-HIV therapy is not curative. We show here that therapy removes the bulk of these immune cell alterations while controlling IFNα levels like EC but with some residual alterations, likely due to IFNα effects before therapy. Importantly, we will also show the cause of the failure of IFNα to contain HIV in the earliest days of infection in non-EC. This leads to a vicious cycle of IFNα response to increasing HIV titers and ultimately to the pathogenic high levels of IFNα. In turn, this leads in non-EC to early immune cell damage, some of which persist even after IFNα control by therapy. Notably, as in most infected patients, anti-HIV therapy did not begin in the earliest days of infection.

## Results

The study seeks to unravel the reason that the anti-HIV effect of IFNα is impaired. As detailed in Methods, we compare: 1) IFNα and IFNλ2 serum concentrations, 2) distribution of immune cell subsets, 3) frequency of cells markers associated with immune dysfunction in treated and untreated infected non-EC patients.

### Distinct blood immune cell profiles and serum IFNα levels in treated patients (TP) compared to untreated patients (UP) and healthy donors (HD).

**Figure 1:**
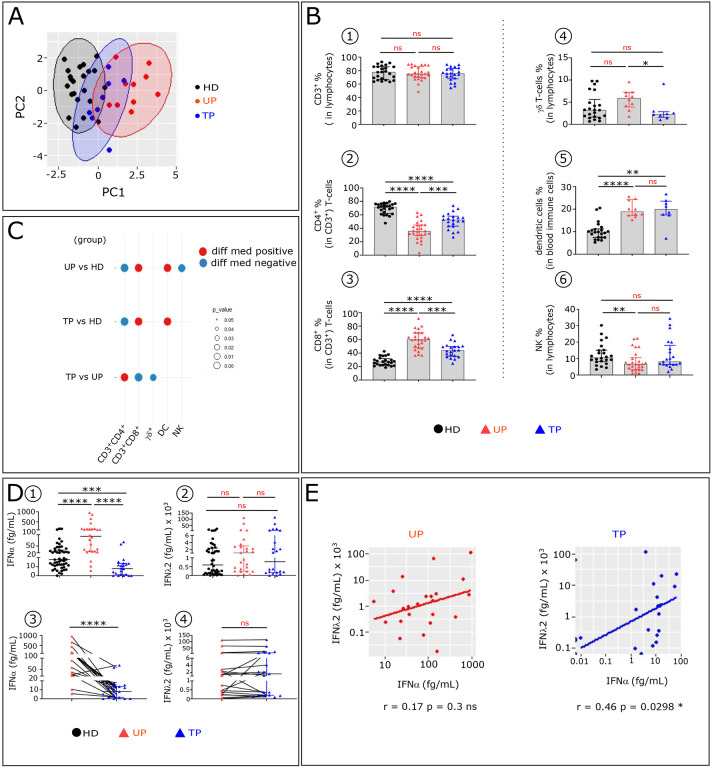
Comparative analysis of major blood immune cell subsets and serum IFNs concentrations in UP, TP and HD. **(A)** Principal component analysis (PCA) of studied participants is based on the proportion of different cell subpopulations (CD4^+^, CD8^+^ and TCR γδ T-cells, NK and DC) evaluated by flow cytometry, as depicted in extended data Fig. 1. The first two Principal components (PC1 and PC2), representing the greatest differences among individuals, are represented on a bi-plot. Each point represents one participant, colored by the group they belong to. Each group is outlined by an ellipse representing the 95% confidence interval of the sample groupings. (**B**) Histograms showing distributions of indicated immune cell populations between HD (Black, n=22), UP (red, n=26), and TP (blue, n=21). (**C**) Balloon-plot summarizing the statistically significant changes in the indicated immune cell populations between UP and HD, TP and HD and TP and UP. The size of the circle represents the p-value. Red and blue colors show increased or decreased frequencies of the immune cell populations respectively. (**D**) Scatter plots showing IFNα and IFNλ2 concentration in serum from HD (n=51), UP (n=26) and TP (n=22). Levels of IFNα and IFNλ2 were detected by SIMOA in unpaired (D1, D2) and paired patients (D3, D4). (**E**) Scatter plot showing relationships between IFNα and IFNλ2 serum levels in the 22 paired UP and TP. Correlations were evaluated with Spearman’s rank correlation test. Differences between unpaired samples and paired samples were performed with Mann-Whitney and Wilcoxon tests respectively. Graph show the median values and p values (*P<0.05, **P<0.01, ***P<0.001, ****P<0.0001)

We first tested whether the major immune cell subtypes frequencies could distinguish TP from UP and HD by principal component analysis (PCA). UP and HD are clearly separated, while TP are distributed between both groups (Fig. 1A). We investigated the immune features that drive this TP immune pattern. No difference in the CD3^+^T-cells frequency are observed between the studied groups (Fig. 1B1). However, like UP, TP show some decrease in CD4^+^T-cells (Fig. 1B2) with a concomitant increase of CD8^+^T-cells (Fig. 1B3), compared to HD. Furthermore, the frequency of γδ T-cells is reduced in TP compared to UP (Fig. 1B4). In addition, the frequency of Dendritic Cells (DC) is increased in TP, like in UP, compared to HD, though to a lesser degree (Fig. 1B5). Finally, the percentage of NK-cells is similar between TP and HD but is markedly reduced in UP (Fig. 1B6). The significant variation of these immune cell types frequencies across the three studied groups is summarized in the balloon plot (Fig. 1C).

We also compared the serum level of IFNα and IFNλ between the three studied groups. TP and HD have highly significant lower serum IFNα level than UP (Fig. 1D1). No remarkable changes in IFNλ concentration between the three groups is observed (Fig. 1D2). The variation of these IFNs in paired samples collected before and after cART confirmed that serum IFNα, but not IFNλ, disappears after treatment (Fig. 1D3–D4 and extended data Table 1). Interestingly, IFNα level is positively correlated with IFNλ2 level in TP as well in EC (14), but not in UP (Fig. 1E).

### High IFNα level enhances *in vitro* expression of the HIV coreceptor CCR5.

**Figure 2:**
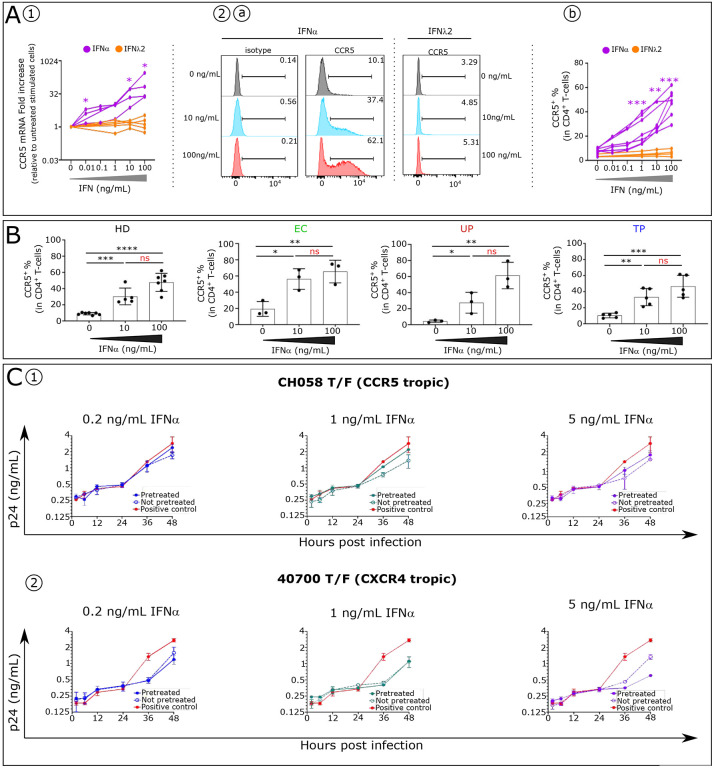
Elevated IFNα effect on HIV coreceptor CCR5 expression in human CD4^+^ T and on release of circulating HIV by infected CD4^+^ T-cells. **(A)** CCR5 expression analysis **(A1)** at the mRNA levels by RT-qPCR and **(A2**) protein levels by flow cytometry in stimulated CD4^+^ T-cells with different concentration of IFNα and IFNλ2 are shown. Differences between unpaired samples were performed with Mann-Whitney test. Graph show the median values and p values (*P<0.05, **P<0.01, ***P<0.001, ****P<0.0001) (**B**) Histograms showing CCR5 frequency in CD4^+^ T-cells stimulated with different IFNα concentrations in each studied group (HD n=7, EC n=3, UP n=3 and post cART n=5). Significance was determined by unpaired Mann-Whitney U test. *P<0.05, **P<0.01, ***P<0.001, ****P<0.0001, ns: not significative. **(C)** PBMCs from a health donor were stimulated in the presence (pretreated) or absence (not pretreated) of IFNα for 4 days. The stimulated cells were infected by the CH058 T/F virus (CCR5 tropic) **(C1)** or the 40700 T/F virus (CXCR4-tropic) **(C2)**. Upon infection, the cells were cultured with corresponding concentration of IFNα for 48 hours. For the positive control, the infected cells (not pretreated) were cultured in the absence of IFNα to determine the normal replication kinetics of the virus. The p24 concentration in the culture supernatants was measured at 2 h, 6 h, 12 h, 24 h, and 48 h post infection. The infections were performed in duplicate, and the error bar represents the standard deviation (SD).

Beta chemokines are the natural ligands of CCR5, and as such are potent inhibitors of HIV (15,16). We previously reported elevated IFNα inhibits the production of these inhibitors of HIV (17). We hypothesized that a greater and more immediate impact would occur if IFNα also directly increased the amount of HIV coreceptor CCR5. Indeed, we show here that IFNα but not IFNλ2 enhances *CCR5* mRNA expression on stimulated CD4^+^T-cells isolated from HD (Fig.2 A1), resulting in higher levels of the CCR5 protein on CD4^+^T-cells (Fig.2A2). Similarly, IFNα induces a dose-dependent increase in the expression of CCR5 on stimulated CD4^+^T-cells isolated from UP, TP and EC (Fig. 2B).

### Upregulation of CCR5 on CD4^+^T-cells by IFNα impairs its anti-HIV effect.

The CCR5 results suggested that the inhibitory effect of IFNα on CCR5 virus replication is compromised, beginning at the onset of infection, and throughout the innate phase of the IR. To test this hypothesis, we infected normal PBMCs with CCR5 (R5) or CXCR4 (X4)-tropic T/F HIV-1 and compared the inhibitory effect of IFNα in IFNα-pretreated and unpretreated cells. For both the CCR5 and CXCR4 viruses, the exponential phase of virus replication was initiated upon 24 hours after infection (Fig. 2C1 and 2C2). For the R5-tropic virus, the level of IFNα inhibition was reduced in IFNα-pretreated cells compared to unpretreated cells. In the cells not pretreated, virus production was inhibited by 39.3%, 51.8% and 49.4% at 48 hours post infection at the concentration of 0.2 ng/mL, 1 ng/mL and 5 ng/mL, respectively (Fig. 2C1). In comparison, in the pretreated cells, the same concentration of IFNα only inhibited virus production by 16.3%, 22.9% and 38.5% at the same time point (Fig. 2C1). As expected for the X4-tropic virus, pretreatment with IFNα had no effect in reducing the level of virus inhibition. In contrast, pretreatment with 5 ng/mL IFNα enhanced the inhibitory effect on the X4-tropic virus (Fig. 2C2). A similar result was observed when PBMCs from a different donor were used (data not shown). These observations indicate that the upregulation of CCR5 by IFNα compromises its inhibition of virus replication for R5 HIV-1, setting the stage for greater damage by both HIV and by a rising level of IFNα.

Considering that IFNα is correlated with HIV load, we assume that during the initial phase of infection elevated IFNα infected CD4^+^T-cells and HIV form a pathogenic vicious circle (HIV → IFNα → infected CD4^+^T-cells → more HIV → more IFNα, etc).

### Persistence of some alterations in immune cell subtypes involved in the innate phase of IR in TP.

**Figure 3:**
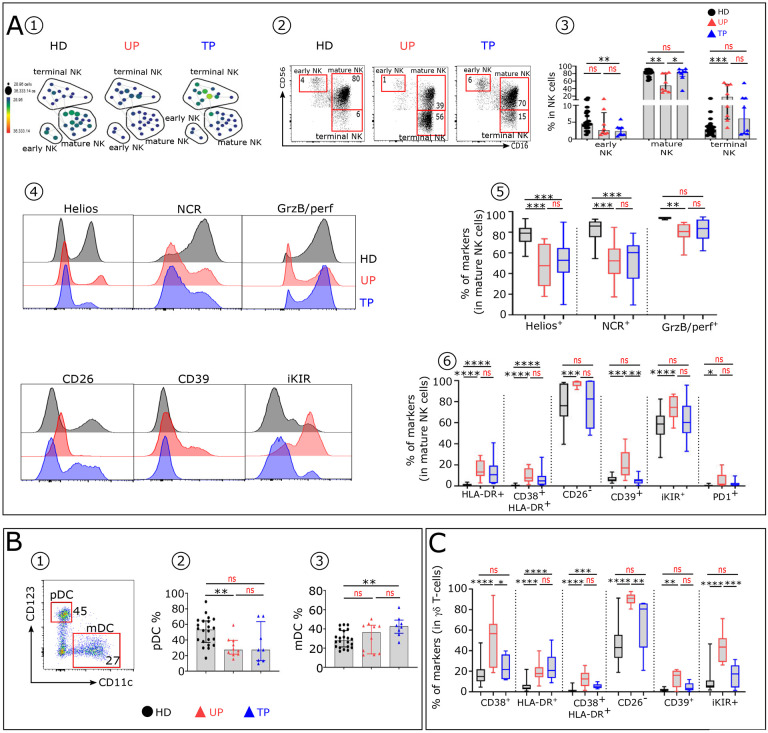
TP distribution analysis of blood innate immune cells show a fewer percentage and fewer phenotypic alterations compared to UP. (**A1**) SPADE tree showing the distribution of NK-cell subsets in HD, UP and TP. Nodes are colored by count. (**A2**) Representative Flow Cytometry plots of NK-cell subsets gated on CD19^−^CD14^−^TCRγδ^−^CD3^−^HLA-DR^−^ cells from the 3 studied groups: early NK (CD56^bright^CD16^−^), mature NK (CD56^dim^CD16^+^) and terminal NK (CD56^−^CD16^+^). (**A3**) Frequency of early, mature, and terminal NK in each studied group (HD n=22, UP n=10 and TP n=8). (**A4**) Histograms displaying the expression level of Helios, NCR (NKp30, NKp44, NKp46), GrzB/perf, CD26, CD39 and iKIR on mature NK cells from HD (black), UP (red) and TP (blue). (**A5** and **A6**) Box plots displaying the frequency of the indicated markers in mature NK-cells in each studied group. **(B1)** Representative dot plot showing how to distinguish pDC (CD123^+^ CD11C^−^) and mDC (CD123^−^CD11C^+^) subsets within the HLA-DR^+^ lin^–^ population in HD. Histograms showing the frequencies of pDC **(B2)** and mDC **(B3)** across the groups (HD n=22, UP n=10 and TP n=8). (**C**) Proportion of specific markers on TCR γδ T-cells of each studied group (HD n=22, UP n=10 and TP n=8). Significance was determined by unpaired Mann-Whitney U test. *P<0.05, **P<0.01, ***P<0.001, ****P<0.0001.

Other than IFNα, components of the innate phase of the IR include particular cells, especially NK-cells. We found differences in the distributions of the 3 major NK-cell subsets (18,19) between UP, TP and HD (Fig. 3A1). The proportion of early NK-cells in UP and TP is lower than in HD. In addition, TP, like UP, but to a lesser extent, show an increase of terminal NK compared to HD (Fig. 3A2–3). We also found that NK-cell subsets from UP and TP display distinct immune profile associated with immune dysfunction. TP mature NK-cells still exhibit lower levels of Helios and NCR and increased levels of IFNα-induced CD38 and HLA-DR (Fig. 3A4–5). In contrast, GrzB, CD26, CD39, iKIR and PD1 expression patterns on mature NK from TP and HD are similar (Fig. 3A4 and 6). We next investigated the distribution of CD11c^+^ mDC and CD123^+^ pDC subsets in the 3 groups (Fig. 3B1). Noteworthy, the pDC frequency is reduced in both patient groups, the decrease being greater in UP, while the mDC proportion is increased in these groups, the increase being higher in TP (Fig. 3B2 and 3B3). The γδ T-cells, another subset of cells of an innate immune response, also show functional signaling receptor expression abnormalities (Fig. 3C) with IFNα-induced HLA-DR, associated in some cases with CD38. These alterations are still significatively more frequent in TP than in HD, but less than in UP.

Collectively, these results show that immune cells from TP, involved during the innate phase of IR, still present functional abnormalities compared to HD, but at a lower level than UP.

### Residual T-cell phenotypic profile abnormalities in TP

**Figure 4:**
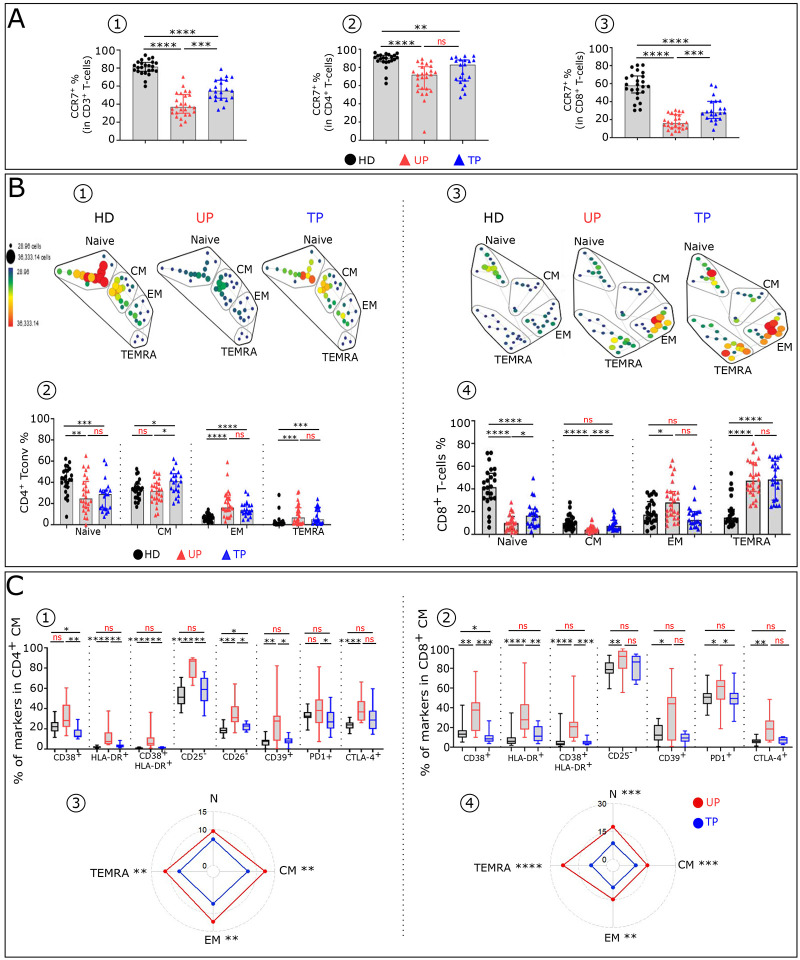
Residual T-cell phenotypic profile abnormalities in TP **(A)** Histograms showing the frequencies of CCR7 in CD3^+^ (**A1**), CD4^+^ (**A2**) and CD8^+^ (**A3**) T-cells across the groups (HD n=2, UP n=26 and TP n=22). (**B**) SPADE tree showing the distribution of CD4^+^ T conv (**B1**) and CD8^+^ T-cell (**B3**) subsets in HD, UP and TP. Nodes are colored by count. CD4 ^+^ Tconv and CD8^+^ T-cells can be classified into four major subsets by their expression of CD45RA and the chemokine receptor CCR7: naïve (CCR7^+^CD45RA^+^); CM (CCR7^+^CD45RA^−^), EM (CCR7^−^CD45RA^−^) and TEMRA (CCR7^−^CD45RA^+^). Frequency of Naïve, CM, EM and TEMRA CD4^+^ Tconv (**B2**) and CD8^+^ T-cells (**B4**) in each studied group (HD n=22, UP n=26 and TP n=22). Boxplots showing the expression of indicated marker in CD4^+^CM (**C1**) and CD8^+^CM (**C2**) across the groups (HD n=22, UP n=10 and TP n=8). Radar chart showing a composite score of phenotypic cell alteration calculated for each CD4^+^ Tconv (**C3**) and CD8^+^ T-cell (**C4**) subpopulations in UP and TP (see Methods). Significance was determined by unpaired Mann-Whitney U test, and correlation with Spearman’s rank correlation test. *P<0.05, **P<0.01, ***P<0.001, ****P<0.0001.

To compare immune T-cell profiles of UP and TP and HD, we characterized the T-cell subtype frequency and phenotypes, as described in material and methods and in the accompanying paper (14). UP have an abnormal T-cell distribution compared to HD. The frequency of CCR7^+^ in CD3^+^, CD4^+^ and CD8^+^T-cells (Fig. 4A1–3) is decreased in UP compared to HD. This loss of T-cells expressing CCR7 is also present in TP, albeit at a lesser degree. UP also exhibit altered proportions of Naïve, CM, EM and TEMRA among CD4^+^ (Fig. 4B1–2) and CD8^+^ (Fig. 4B3–4) T-cell subsets compared to HD. TP still show an abnormal differentiation profile but with a normalization of their CM and EM CD8^+^ frequency. In addition, UP exhibit an altered phenotypic profile in T-cell subsets, compared to HD (Fig. 4C). This can be partly attributed to high IFNα levels (14). These phenotypic alterations are characterized by an abnormal level of activation/differentiation markers (CD38, HLA-DR, CD25, CD26, CD28) and inhibitory receptors (CD39, PD1, CTLA4) (Fig. 4C and extended data Fig. 2). By and large, in contrast to UP, TP display similar immune profiles to HD. However, in TP, we still identified phenotypes in CD8^+^T-cells, such as HLA-DR^+^CM, CD38^+^HLA-DR^+^EM, which remain significantly different to HD. The phenotypic analysis shows that after treatment partial reversion of T cell phenotype anomalies in all CD4^+^ and CD8^+^T-cell subsets is observed, as evaluated by the phenotypic alterations scores (see Methods) (Fig. 4C3–4). Interestingly, in UP, the expression levels of various markers associated with immune dysfunction in CD4^+^ and CD8^+^ CM population are directly correlated (extended data Fig. 2A4 and 2B4). This is in keeping with the interpretation that these are alterations induced by one major mediator such as elevated IFNα. No such correlation is found after therapy.

**Figure 5:**
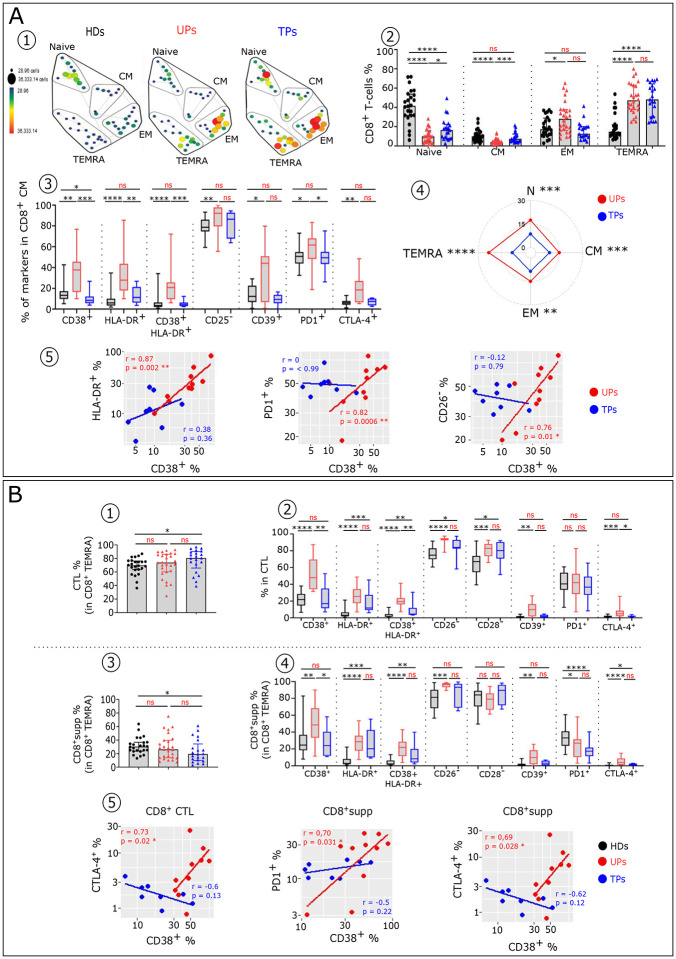
Treg and cytotoxic CD8^+^ T-cells from TP exhibit residual phenotypic abnormalities. (**A1**) Representative flow cytometry plots of CD25^+^ Foxp3^+^ cells within CD4^+^ T-cells isolated from HD, UP and TP. (**A2**) Histograms showing the frequency of Foxp3 in CD4^+^ T-cells. (**A3**) Histograms displaying the expression level of CD25 in CD4^+^ Foxp3^+^ T-cells and (**A4**) the frequency of Treg CD25^−^ variant in CD4^+^Foxp3 T-cells in each studied group. **(A5)** Proportion of specific functional signaling checkpoints on memory CD4^+^ Treg (CD4^+^ Foxp3^+^CD25^+^ CD45RA^−^) of each studied group (HD n=22, UP n=10 and TP n=8). (**B**) Histograms showing the frequency of CD8^+^ CTL (CD8^+^TEMRA iKIR^−^ (**B1**) and CD8^+^supp (CD8^+^TEMRA iKIR^+^) (**B3**) in each studied group (HD n=22, UP n=26 and TP n=22). Box plots showing the proportion of specific markers on CD8^+^ CTL (**B2**) and CD8^+^supp (**B4**) T-cell subsets of each studied group (HD n=22, UP n=10 and TP n=8). (**B5**) Scatterplots showing relationships between the expression level of indicated markers in the CD8^+^ cytotoxic T-cells (CD8^+^ CTL and CD8^+^supp) (UP n=10 and TP n=8). Significance was determined by unpaired Mann-Whitney U test, and correlation with Spearman’s rank correlation test. *P<0.05, **P<0.01, ***P<0.001, ****P<0.0001.

The Treg phenotypic profile analysis revealed an increase frequency of Treg in UP compared to HD (Fig. 5A1–2). This increase in Treg frequency is, however, not seen in TP. In contrast, we found that after treatment, patients still have a high frequency of Treg lacking CD25 expression (20), and the frequency of this CD25^neg^ Treg variant is higher in UP (Fig. 5A3–4). However, the altered immune profile of memory Treg observed in UP is partially restored in TP (Fig. 5A5).

As to the CD8^+^T-cell subsets, quantitative and qualitative defects of differentiated CTL (KIR^−^) and CD8^+^supp (KIR^+^) among the TEMRA subset were found in HIV-infected patients (Fig. 5B). We found an increase of CTL with a concomitant reduction of CD8^+^supp in TP compared to HD (Fig. 5B1 and 5B3). These populations in UP and HD are similar. In addition, UP show a higher frequency of CTL and CD8^+^supp cells expressing various markers of activation, differentiation and exhaustion compared to HD, while TP display almost similar phenotypic pattern to HD (Fig. 5B2 and 5B4). We also found a correlation between IFNα-induced CD38 and expression of inhibitory checkpoints (CTLA-4 and PD1) in both CTL and CD8^+^supp in UP, but not after therapy (Fig. 5B5).

In summary, the phenotypic anomalies associated with loss of immune cell function of Tconv and Treg cells is markedly reduced in TP compared to UP, though they are of similar nature as in UP, and in large part these are proteins induced by IFNα. The CD8^+^ functionally-linked phenotypic alterations are also of the same nature as found in CD4^+^T-cells, but far more, and are markedly reduced after therapy. These immune alterations in both Tconv and CD8^+^ cytotoxic T-cells observed before therapy and still present after cART may account for the fragile health indicated by the comorbidities that still occur in the TP.

### TP and EC exhibit similarities in immune profiles

**Figure 6:**
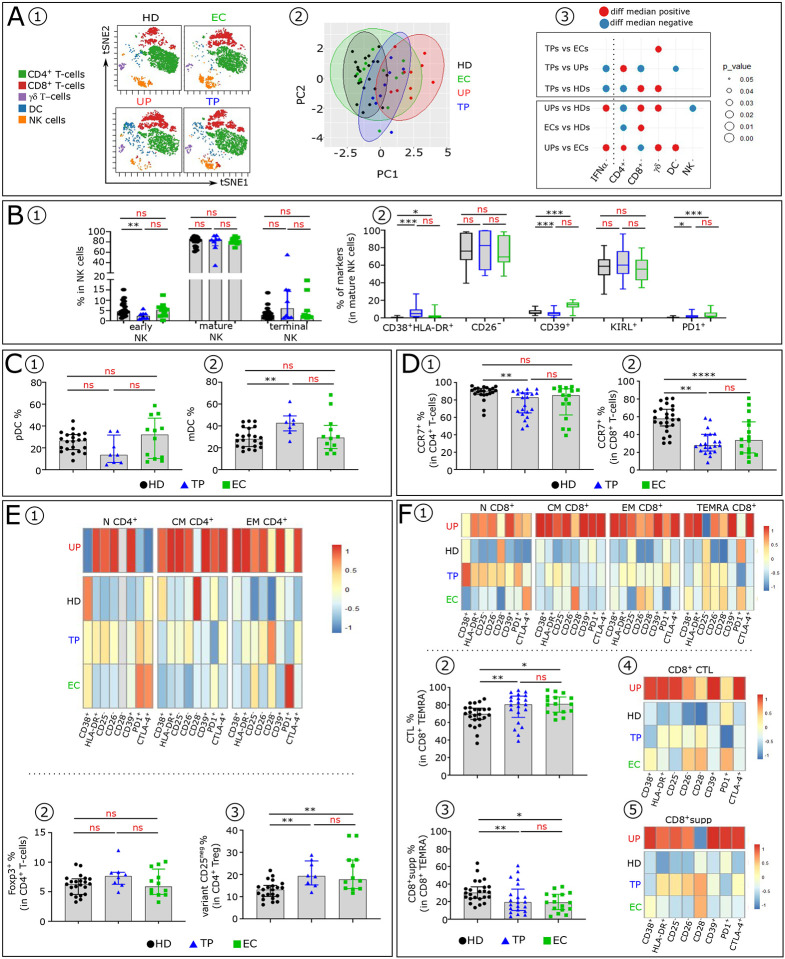
TP and EC share few blood immune cell anomalies. (**A1**) Representative viSNE plot showing major immune cell subpopulations distribution (CD4^+^, CD8^+^ and TCR γδ T-cells, NK and DC) in HD, UP, TP and EC, evaluated by flow cytometry. (**A2**) PCA scatter plots of samples based on proportion of the different major lymphocyte subpopulations indicated above. Each group is outlined by an ellipse representing the 95% confidence interval of the sample groupings. (**A3**) Balloon-plot summarizing the statistically significant changes in the indicated immune cell populations between each compared group. (**B1**) Frequency of early, mature and terminal NK in each studied group (HD n=22, TP n=8 and EC n=12). (**B2**) Box plots displaying indicated markers frequency in mature NK-cells in each studied group. Histograms showing the frequencies of pDC **(C1)** and mDC **(C2)** across the groups (HD n=22, TP n=8 and EC n=12). Histograms showing CCR7 frequency in CD4^+^ (**D1**) and CD8^+^ (**D2**) T-cells across the groups (HD n=22, TP n=8 and EC n=12). (**E1**) Heatmap showing indicated markers frequency in CD4^+^N, CM and EM. (**E2**) Histograms showing Foxp3 frequency in CD4^+^ T-cells and (**E3**) Treg CD25^−^ variant frequency in CD4^+^Foxp3 T-cells in each studied group. (**F1**) Heatmap showing indicated markers frequency in CD8^+^N, CM EM and TEMRA. Histograms showing the frequency of CD8^+^ CTL (**F2**) and CD8^+^supp (**F3**) in each studied group (HD n=22, TP n=8 and EC n=12). Heatmap showing indicated markers frequency in CD8^+^ CTL (**F4**) and in CD8^+^supp (**F5**) in each studied group (HD n=22, TP n=8 and EC n=12).

We next analyzed the cell blood immune profiles in TP compared to EC (Fig. 6). VISNE and PCA analysis indicate that TP and EC display similar cell distribution distinct from UP and HD (Fig. 6A1–2). Both TP and EC also have normal background levels of serum IFNα. One difference between them, occurs with γδ T-cells (Fig 6A3). No difference was found in the proportion of the NK-cell subsets among TP and EC, whereas a decrease of early NK-cells is observed in TP compared to HD (Fig. 6B1). Analysis of phenotypic abnormalities in mature NK-cells showed a higher frequency of CD38^+^ HLA-DR^+^ and PD1^+^ cells in TP and EC than in HD (Fig. 6B2). Interestingly, TP and EC also share a similar percentage of DCs (Fig. 6C1–2). One difference observed in the DC compartment is a significant increase of mDC subset in TP.

Dysregulation of T-cells homeostasis, observed in untreated HIV-1 infected patients (21), is maintained in TP, albeit to a lesser degree, than in EC. TP have less CCR7^+^ CD4^+^T-cells than HD (Fig. 6D1). In CD8^+^T-cells, TP and EC have decreased frequencies of CCR7^+^T-cells compared to HD (Fig. 6D2). Furthermore, the few T-cell phenotypic alterations of TP and EC are similar (Fig. 6E1 and 6F1 and extended data Fig. 3). Finally, analysis of Treg cells (Fig. 6E2–3) revealed that their frequencies are similar in the three groups (Fig. 6E2). Noteworthy, TP and EC have an increase in the CD25^neg^ Treg variant compared to HD (Fig. 6E3). Within CD8^+^TEMRA, TP and EC show an increase of CTL with concomitant decrease of CD8^+^supp compared to HD (Fig. 6F2–3). The anomalies observed in TP and EC are much less pronounced than in UP but slightly higher than in HD (Fig. 6F4–5 and extended data Fig. 3).

Collectively these data show that as opposed to UP, TP and EC do not express abnormal high serum IFNα level, avoiding the pathogenic IFNα effects on immune cells. Particularly notable were the marked improvement of the early effective NK-cells and the later developing effective antigen specific HLA-1 restricted CD8^+^ CTL and HLA-E restricted CD8^+^supp in TP and EC. However, they do not completely return to the status of HD.

## Discussion

The advances in therapy have derived from understanding the molecular events of HIV replication and targeting one or more stages with specific drugs. However, therapy is required lifelong except for EC. Except for developing long-lasting therapies and perhaps targeting the integrated HIV proviral DNA of HIV-reservoir cells, we need alternative approaches. In agreement with the EC consortium (13) we think a deeper penetration of HIV pathogenesis and learning how EC avoids these pathogenic mechanisms may be key to future advances in which the >99% of infected non-EC patients can be converted to an EC status without treatment and possibly functionally cured.

There is an abundance of evidence showing that the key events leading to HIV progression and AIDS or a favorable clinical outcome are predicated on the events of early infection (22,23). What that early damage is and its prognostic underpinning have been unclear. Its first signs are the HIV peak viremia and subsequent virus set point. These occur during the very initial stage of the immune response, the time of innate immunity. If not kept under tight control, a high HIV level leaves its stamp on future developments. We have known that HIV kills infected CD4^+^T-cells, chiefly occurring after antigen activation (24). Though a small percentage of macrophages are also infected, most occur without cytopathic effects (25), and other immune cells, are not infected. It was evident, however, that there is much more to the story, and many reports show some other uninfected immune cells are not functioning normally. This suggests there is one or more unidentified critical mediator(s) of these effects, which is very early, thereby excluding specific adaptive immune responses, and not from the later inflammatory cytokines of the chronic stages. Instead, this mediator must be active at the time of the innate stage of the immune response (extended data Table 2).

Our results and the literature lead us to conclude that the main mediator is elevated levels of IFNα (14). Negative aspects of IFNα have been described, but usually as one of several cytokines and acting in some general way to promote inflammation as occurs in later stages. The details in our reports show that IFNα is more important, early and specific. Another contributor to innate immunity are NK-cells. Greater than 99% of infected persons do not have adequate early control of HIV. As such, HIV progression has its tailwind. As we show in both the accompanying paper and in this report, both NK-cells and IFNα show major abnormalities after infection in UP. NK-cells remain unharmed in EC (14) and markedly improved in non-EC after therapy (shown here). In these patients, NK-cells show particular features of normal NK-cells, especially in the setting of HLA-B57. Undoubtedly, NK-cells are a key factor in the control of HIV in the earliest days after infection. IFNα levels are highly elevated from an early period in UP, and it is directly correlated with the HIV titer (26,27). In the specific case of HIV, IFNα is not a very effective antiviral since it concomitantly induces high expression of the HIV co-receptor, CCR5, as we show here (Fig. 5 and extended data Table 2) and as we previously reported also reduces the HIV inhibitory beta chemokines (17). We demonstrate that in untreated HIV infected non-EC patients all peripheral blood immune cells and at all stages of an immune response show abnormalities of distribution numbers and immune phenotype. Importantly, these abnormalities can be induced by elevated IFNα levels, as described in the companion paper or in a few specific cases reported by us and others (28,29, 30). They include induction of several inhibitory checkpoints and immunosuppressive molecules, diminishing some essential positive immune signals, and interference with T-cell homeostasis and the subsequent adaptive immune response.

We show that EC and, to a lesser extent TP avoid these abnormalities of immune cell subsets (Figs. 2–5), and control IFNα levels and virus load (Fig. 1). The differences in immune cells anomalies between TP and UP are huge. Apart from the control of IFNα production, in TP and EC, these particularly include : 1) fewer infected CD4^+^T-cells likely due to the absence of IFNα-induced enhancement of CCR5 (Fig. 5); 2) the presence of functional NK-cells lacking any major IFNα-induced immune defects (Fig. 3A); 3) low levels of IFNα-induced CD4^+^ T helper and regulatory cells alteration (Fig.3) and 4) minimally altered antigen specific cytotoxic CD8^+^T-cells (both CTLs and CD8^+^supp T-cells) which also lack the major IFNα-induced defects (Fig. 4). We suggest that the remaining defects in EC and in TP are the residue of the early phase of the IR due to the known thymic damages, whether IFNα-induced or not (extended data Table 2) and further in TP before cART (31,32).

IFNλ is a different story. Even in TP, IFNλ remains elevated suggesting persistence of local mucosal tissue reservoirs of HIV. However, IFNλ does not have detrimental effects on immune cells like IFNα because immune CD4^+^T-cells do not have constitutive receptors for IFNλ (33), nor does it enhance CCR5 expression (Fig. 2A). Consequently, IFNλ may be a useful agent against HIV even when present at high levels.

Evidence indicates that some EC are partially protected by genetic factors, particularly their HLA genetics, and most especially HLA-B57 (34). Indeed, it is tempting to think that these are the answers for explaining the EC state because genetic factors are, of course, present at the onset of infection, and the evidence dictating progression strongly favors an early event. However, for explaining the entirety of the EC group, this cannot be true because known genetic factors are not present in many EC, and HLA-B57 is present in many HIV non-EC who demonstrate typical disease progression (34). A reduced CCR5 genetic trait has also been described but present only in a small number of EC (35). Our data argue against a single trait for becoming an EC because their minimal abnormalities are numerous and diverse. Consequently, we have hypothesized that there may be an additional mechanism, which would also be at the onset: a fortuitous infection with a low inoculum of HIV. This would reduce the number of competing founder viruses and favor chances for a lower producer. From the onset, this would mean for EC low HIV input → low set point → low IFNα → no negative impact on immune cells control of HIV. Though a difficult and long-term challenge, this idea is testable in a few ways. First, it predicts a substantially longer eclipse time. This period (time of infection to detectable viremia) is about two weeks as shown by M. Robb and colleagues in HIV-infected non-EC patients (36). The hypothesis predicts a significantly longer time in EC. Second, we may be able to reproduce the proviral integration pattern of EC with low dose infection in animal models. Third, patients infected with larger inoculums such as hemophilia patients infected in the early years of HIV, should not become EC.

HIV/AIDS is now a treatable disease, and for >99% of patients, therapy is a lifelong, sometimes with side effects, co-morbidities, and possibly a shortened life span. A goal in the field is to develop a functional “cure” without needing further therapy, but recognizing that some HIV cell reservoirs with “silent” proviral DNA may remain that can be re-awakened with therapy interruption or by external stimuli. Consequently, something more is needed. It is unlikely that many novel standard approaches, based on our detailed knowledge of the molecular events in HIV replication, are left for us to explore other than longer-lasting drugs and perhaps specific attacks on HIV proviral DNA. In agreement with the EC consortium (13) and its leadership, we suggest it may be rewarding in the quest for functional cure to focus on pathogenesis with an eye on therapies that diminish HIV progression and imitate the EC state. In this regard, the work of the consortium defines a unique pattern of HIV integration, including spaced mono/oligoclonal clusters and HIV proviral DNA integration in silent DNA regions with a reduced frequency of escape mutations in cytotoxic epitopes and antibody contact regions (13,37), and they have suggested therapies geared toward reproducing this pattern in non-EC. We suggest that this pattern of HIV proviral integration may be an event not originally causing the control of HIV but rather the consequences of HIV control, but which nonetheless subsequently helps maintain that control by markedly diminishing HIV expression. The key question then is what is this original control? We hypothesize and our data indicate that it is the prevention of elevated IFNα in the earliest stages, whether through immediate therapy or by a low inoculum initiating infection.

To conclude, IFNα, of course, is a key and an immediate protector against foreign invaders. Sometimes it is also a well-known contributor along with several other cytokines to dangerous inflammation in the later stages of infection by some viruses. However, our results here are directed to a new and an early as well as continuous *solo* pathogenic effect. These results place elevated IFNα as a key direct mediator of HIV pathogenesis, in addition to the known direct HIV effect. The IFNα effect occurs along with HIV early after infection and continues to AIDS progression unless treatment is initiated, or in the case of EC, as we hypothesize here, can be avoided because of low inoculum infection. We emphasize here, that, beside ART, therapy should include temporarily targeting elevated systemic IFNα, as soon as HIV seropositivity is known, in order to reduce measurable HIV proviral DNA intact sequences down to the EC level (14,37), while concomitantly providing unharmful anti-viral IFNα to control other viral infections and hopefully this approach will lead to the lack of the further need for ART, ie a functional cure, as a direction promoted by B. Walker and colleagues (13).

## Online Methods

### Human samples.

HD were obtained through Etablissement Français du Sang (EFS, Paris, France). 67 people living with HIV were recruited and subdivided into three group: EC (n=18) were obtained from the NVS cohort (Baltimore), UP (n=36) were obtained from NIH (Bethesda n=19) and from the Laboratoire de Référence SIDA (Liège n=17) and TP (n=27) from NIH (Bethesda n=19) and from Laboratoire de Référence SIDA (Liège n=8). Patient group did not significantly differ in terms of age, gender, disease status. All participants or their surrogates provided informed consent in accordance with protocols approved by the regional ethical research boards and the Declaration of Helsinki. Clinical data are indicated in extended Table 3a–c.

### Sample processing.

Peripheral blood and serum were collected into appropriate tubes. PBMCs were isolated by density gradient centrifugation on Ficoll-Hypaque (Pharmacia, St Quentin en Yvelines, France. PBMCs were stored frozen in liquid.

### T-cells culture.

CD4^+^ T cells were isolated from frozen PBMCs. All CD4^+^ T cells were positively selected with a CD4^+^ T cell isolation kit (Miltenyi Biotec, Bergisch-Gladbach, Germany), yielding CD4^+^ T cell populations at a purity of 96–99%. Purified CD4^+^ T-cells were stimulated as described previously (20) with 4 μg/mL plate-bound anti-human CD3 (OKT3) mAb (eBioscience, San Diego, CA) and 4 μg/mL soluble anti-human CD28 (CD28.2) mAb (Becton Dickinson) in presence of Recombinant human IL-2 (Proleukine, Chiron, Amsterdam, 100 U/mL) and recombinant human interferon alpha-2a (Roferon-A 0.01–100 ng/mL). After four days culture, membrane CCR5 expression was measured by flow cytometry on CD4^+^ T cells.

### Infectious molecular clones.

The infectious molecular clone (IMC) of the CH058 transmitted/founder (T/F) virus (CCR5-tropic) was obtained from the NIH HIV Reagent Program. The CXCR4-tropic T/F virus 40700 which does not have CCR5-using ability was identified in an acute CRF01_AE infection. The full-length genome of the 40700 T/F virus was determined using single-genome amplification (SGA) as previously described (38). The detailed information of the 40700 IMC, including the full-length viral sequence will be described in a separate manuscript.

### Viral stock preparation and titration.

To generate viral stocks, 6 μg of IMC was transfected into 293T cells in a T25 flask using the FuGENE6 transfection reagent (Promega). Six hours post transfection, the culture medium containing the plasmids and the transfection reagent was completely replaced with 8 mL of fresh medium. The cells were cultured at 37°C for 3 days. The culture supernatants were harvested at 72 hours post transfection, filtered, and stored at −80°C until use. The infectious titers (TCID50) of the viral stocks were determined on TZM-bl cells.

### Determination of virus growth kinetics in PBMCs.

Cryopreserved PBMCs from a health donor were thawed and recovered overnight at 37°C in RPMI1640 containing 10% FBS. The next day, the cells were stimulated with 4 μg/mL plate bounded anti-CD3 (clone OKT3, eBioscience) and 4 μg/mL soluble anti-CD28 (clone CD28.2, eBioscience) in the presence of 100 U/mL IL-2 (Human IL-2 IS, Miltenyi Biotec) in a 96-well U bottom plate at a density of 0.3 × 10exp6 cells/well. To determine whether the upregulation of CCR5 by IFNα could compromise its inhibitory effect on HIV-1 replication, the cells were pretreated with three different concentrations of IFNα (0.2 ng/mL, 1 ng/mL, and 5 ng/mL) during the stimulation step. Cells stimulated in the absence of IFNα (not pretreated) were used as a control. Four days after stimulation, the cells were washed twice to remove the stimulating antibodies and infected by the CH058 or 40700 T/F virus with a multiplicity of infection (MOI) of 0.05. After 4 hours infection at 37°C, the cells were washed three times with RPMI1640. The infected cells were cultured in a 48-well plate with 500 μL RPMI1640 containing 10% FBS, 100 U/mL IL-2 and corresponding concentration of IFNα (0.2 ng/mL, 1 ng/mL, and 5 ng/mL). As a positive control, the infected cells (not pretreated) were cultured in the absence of IFNα to determine the normal replication kinetics of the virus. The culture supernatants were harvested at 2 h, 6 h, 12 h, 24 h and 48 h post infection, and virus replication was determined by measuring the p24 concentration in the supernatants.

### Antibody panels, staining and flow cytometry analysis.

**Extended data Fig. 1:**
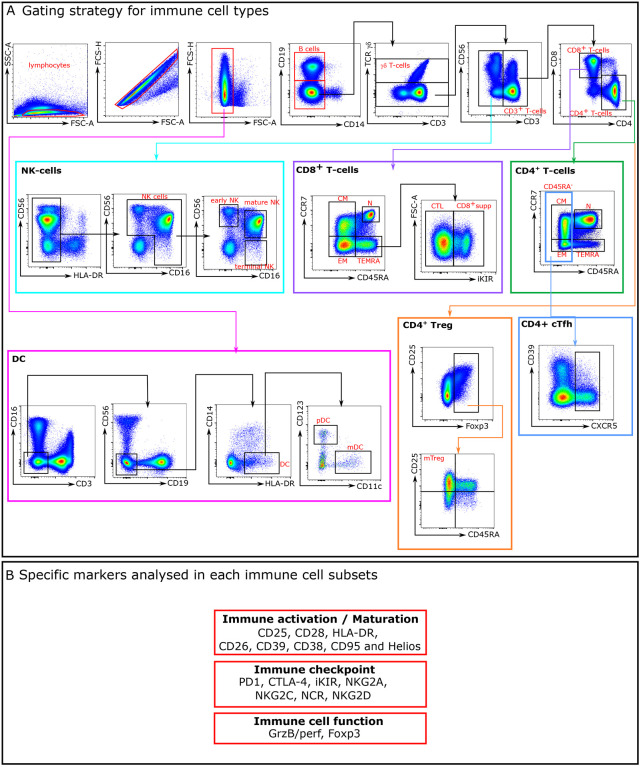
Gating strategy for immune cell types. A- Gating strategy for immune cell types. The gating strategy used to identify the main cellular subsets is presented. Arrows are used to visualize the relationships across plots, and numbers are used to call attention to populations described here. After doublets and dead cells were excluded, lymphocytes were gated based on FSC^−^A/SSC^−^ A properties. From the CD14^−^CD19^−^ lymphocyte gate, the following populations were identified: CD3^+^TCRγδ^+^, TCRγδ^−^ were subdivided in CD3^−^ and CD3^+^ T-cells. NK cells were defined as CD3−TCRγδ−HLA^−^DR−and classified as early NK (CD56^+^CD16−), mature NK (CD56^+^CD16^+^), and terminal NK (CD56−CD16^+^) cells. The CD3^+^TCRγδ− population was divided in CD4^+^ and CD8^+^ T^−^cells. In CD4^+^ T^−^cells subpopulation, CCR7^+^ and CD45RA^+^ were used to further classify these cells in four subpopulations: N (CCR7^+^CD45RA^+^), CM (CCR7^+^CD45RA^−^), EM (CCR7^−^CD45RA^−^) and TEMRA (CCR7^−^CD45RA^+^). Tregs were identified from the CD4^+^ population using Foxp3 expression. Foxp3^+^ cells were classified in naïve and memory Treg cells using CD45RA and CD25 markers. CD45RA^−^CD25^+^ represent the memory Treg cells population. As for CD4^+^ T-cells, CD8^+^ T-cells were classified using CD45RA and CCR7 markers: four populations were identified: N (CCR7^+^CD45RA^+^), CM (CCR7^+^CD45RA^−^), EM (CCR7^−^CD45RA^−^) and TEMRA (CCR7^−^CD45RA^+^). Among TEMRA CD8^+^ T^−^cells, we distinguished two cytotoxic subpopulations: iKIR^+^ (CD8^+^supp) and iKIR^−^ (CTL). Dendritic cells (DCs) were identified by gating on CD3^−^CD19^−^CD56^−^CD14^−^HLA^−^DR^+^ and from there CD123^+^CD11c^−^ (pDCs) and CD11c^+^CD123^−^ mDCs were identified. B- Specific markers analysed in each immune cell subsets.

Immunophenotypic studies were performed on frozen samples, using up to 23-colours flow cytometry panels. See extended Tables 4–6 for antibody panel information. The list of mAbs used are detailed in gating strategy we used to identify the immune cell subtypes and their respective subsets are represented in extended data Fig. 1. Antibody titration was performed to choose the concentration that provided the maximal brightness of the positive cell population and the lowest signal for the negative cell population. Approximately 1 × 10^6^ to 5 × 10^6^ frozen PBMCs were used per patient per stain. Staining was performed as described previously (20). Cells were acquired on Cytek Aurora flow cytometer. Data were analysed using FlowJo software (FlowJo, LLC). Unsupervised analyses were performed using cytobank software and R studio software.

### Composite cell phenotypic alteration score.

We generate a cumulative phenotypic score for each T-cell subsets. The frequency of the following markers was used to calculate the score: CD25^−^, CD26^−^; HLA-DR^+^, CD38^+^, CTLA-4^+^, CD28^−^ , PD1^+^ and CD39^+^. It is calculated as the sum of the ratio of the expression level of each marker to the average expression level of the corresponding marker in the HD.

### Cytokines quantification.

Serum IFNα and IFNλ2 levels were determined using Simoa cytokine assays (references 100860 and 101419 respectively). Van der Sluis et al showing that HIV infection of co-cultures of CD4+ T cells and pDCs enhanced mRNA expression of IFNλ2 and not IFNλ1 or IFNλ3, we focused only on serum IFNλ2 levels (39).

### Statistical analyses.

Statistical significance of differences between group was assessed using the unpaired nonparametric Mann-Whitney. Non-parametric, paired Wilcoxon tests were used for paired data. Correlations were assessed by the nonparametric Spearman test. Analyses were performed with GraphPad-Prism, and R. Two-sided P value less than .05 was considered statistically significant (ns: nonsignificant; *P < .05; **P < .01; ***P < .001; ****P < .0001).

## Extended Data

**Extended data Table 1:** IFNα and IFNλ paired-sera concentrations variations from individual patients collected before (pre-cART) and after (post-cART) treatment.

Pre-cART	Post-cART	VL (particle/ml)		CD4 count (/ml)		INFα (fg/ml)		IFNλ (fg/ml)	
4	11	132773	<40	327	744	958.93	4.17	112328	115318
SS_21	SS_31	3012	<50	292	402	659.46	58.34	10424	9082
3	15	153719	na	230	na	441.17	0	371	197
SS_23	SS_33	<50	<50	639	603	161.74	7.62	17	114
SS_26	SS_36	11417	<40	740	1328	156.71	11.74	2868	4080
6	16	12015	<40	513	na	138.01	0	65726	63032
SS_27	SS_37	63954	<40	344	410	128.63	12.39	1593	2166
SS_29	SS_39	17809	<50	196	437	127.82	5.07	451	365
SS_22	SS_32	29257	<40	477	627	119.62	13.48	2284	1192
5	17	1715	<40	419	na	113.4	10.9	216	152
2	12	58059	<40	516	na	90.02	0.01	2337	210
SS_24	SS_34	26059	<50	267	404	58.46	8.03	1139	10548
SS_28	SS_38	7180	<40	872	682	36.6	15.3	472	2227
7	18	21701	<40	447	na	26.73	13.82	755	352
SS_20	SS_30	407	<50	445	503	26.1	68	12874	22199
SS_25	SS_35	5384	<50	470	459	23.76	17.79	244	19634
9	14	7011	<40	756	809	22.64	0	56	174
1	13	23808	<40	506	685	10.52	1.49	238	93
8	10	4785	<40	319	na	5.65	1.63	1430	1627
HD (med) n=65		none		na		17.7		595	
pré cART (med) n=19		14912		447		113		1139	
post cART (med) n=19		<40		603		8.03		1627	
EC (med) n=18		114		878		15.27		672	

This table presents individual serologic levels of IFNα and IFNλ from HIV patients of the tested cohort, before (red) and after treatment (blue), the viral loads and CD4^+^ T-cell counts are also included. The list is sorted by decreasing IFNα values in untreated patients. (na : non available)

**Extended data Table 2:** IFNα the key mediator of HIV pathogenesis

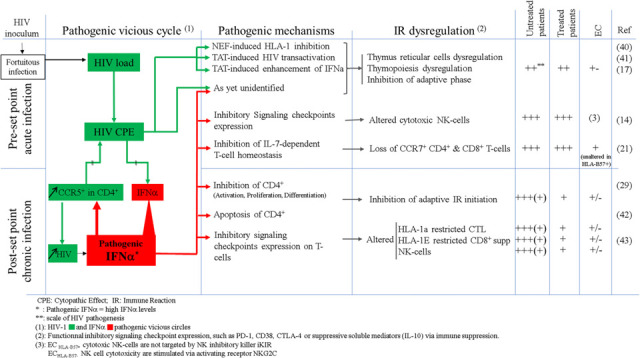

**Extended data Table 3:** Clinical data for HIV patients

*Table 3a : EC patients*								
EC	HIV dx	Sample date	CD4 at sample date	VL	HLA B57	Sex	Race	HIV risk factor
3	1991	10/12/012	1140	334	−	M	AA	IDU
4	1993	22/08/2011	952	<40	+	F	AA	HS
6	1992	31/05/2011	1889	<75	+	F	AA	IDU
8	1989	01/02/2010	1018	<40	+	M	AA	IDU
9	2003	25/09/2014	496	<40	+	M	AA	IDU
11	1997	10/72/011	917	<40	+	M	AA	IDU
13	1993	21/02/2012	864	<40	−	M	AA	IDU
31	1992	23/05/2012	587	<48	−	M	AA	MSM
32	2000	23/05/2012	643	<48	−	F	AA	IDU
42	1990	13/11/2013	731	<220	+	M	AA	IDU
47	2007	01/12/2013	891	<20	−	F	AA	IDU
51	2007	20/10/2014	1250	<40	+	F	AA	HS
52	1992	01/08/2012	340	<20	+	M	AA	IDU
55	1994	01/02/2013	482	50	−	M	AA	IDU
58	1991	01/03/2013	1745	59	−	F	AA	HS
63	1988	24/01/2014	632	32	+	F	AA	IDU
65	1991	01/06/2013	1792	<48	+	M	AA	HS
68	2002	01/07/2010	584	169	+	F	AA	HS

*Table 3b: HIV untreated patients*				
Untreated patients study_ID	sample date	CD4 at sample date	VL	Origin
**1**	03/11/2004	506	23808	NIH (Bethesda)
**2**	22/04/2004	516	58059	
**3**	01/10/2005	230	153719	
**4**	27/09/2005	327	132773	
**5**	19/06/2006	419	1715	
**6**	04/10/2007	513	12015	
**7**	13/08/2007	447	21701	
**8**	13/03/2008	319	4785	
**9**	05/11/2011	756	7011	
**SS_20**	08/10/2005	445	407	
**SS_21**	11/10/2009	292	3012	
**SS_22**	10/07/2010	477	29257	
**SS_23**	07/22/08	639	<50	
**SS_24**	11/17/2008	267	26059	
**SS_25**	03/23/2006	470	5384	
**SS_26**	11/07/2012	740	11417	
**SS_27**	01/28/2014	344	63954	
**SS_28**	11/04/2010	872	7180	
**SS_29**	12/03/2002	196	17809	
**AZT70934**	12/03/2021	470	2480	Laboratoire de Référence SIDA (Liège)
**AZT71312=1373**	12/03/2021	100	24800	
**AZT_71320_AFCHM_6**	12/08/2020	139	32800	
**AZT_71333_KWGIF_7**	12/09/2021	356	9790	
**AZT_71374_DIAMM_8**		390	588000	
**AZT_71391_GAFUM_9**	02/24/2021	1064	14700	
**AZT71422**	03/05/2021	30	850000	
**AZT_72336_GEMAM_11**		20	824	
**AZT_72498_YOLEF_12**		280	82700	
**AZT_72607_CHUIN**	03/16/2021	577	157000	
**AZT_72644_NGNNF**	03/18/2021	180	73700	
**AZT_76144**	01/21/2022	321	122000	
**AZT_76469**	02/18/2022	489	40000	
**AZT_76532**	02/24/2022	449	1420000	
**AZT_7525**	12/16/2021	405	49800	
**AZT_75872**	12/30/2021	260	80900	
**AZT_74040**	0719/2021	739	20300	

*Table 3c: HIV treated patients*				
Treated patients study ID	sample date	CD4 at sample date	VL	Origin
**13**	02/10/2017	685	<40	NIH (Bethesda)
**12**	1/19/2017	912	<40	
**15**	11/27/2017	NA	na	
**11**	12/08/2016	744	<40	
**17**	7/19/2018	NA	<40	
**16**	4/13/2018	NA	<40	
**18**	9/26/2018	NA	<40	
**10**	03/07/2012	417	<40	
**14**	3/27/2017	809	<40	
**SS_30**	08/10/2006	503	<50	
**SS_31**	05/09/2012	402	<50	
**SS_32**	04/02/2013	627	<40	
**SS_33**	04/30/2010	603	<50	
**SS_34**	08/25/2010	404	<50	
**SS_35**	10/15/10	459	<50	
**SS_36**	10/21/2014	1328	<40	
**SS_37**	05/13/2016	410	<40	
**SS_38**	08/06/2010	682	<40	
**SS_39**	08/19/2004	437	<50	
**AZT_72855_1**	04/12/2021	245,6	<20	Laboratoire de Référence SIDA (Liège)
**AZT_72856_2**	04/12/2021	0	<20	
**AZT_72979_3**	04/21/2021	31,65	<20	
**AZT_70944**	10/27/2020	869	<20	
**AZT_73954**	07/13/2021	1016	<20	
**AZT_73955**	07/13/2021	788	<20	
**AZT_73989**	07/14/2021	960	<20	
**AZT_74100**	08/28/2021	1530	<20	

**Extended data Table 4:** mAb list for immune cells panel.

	Markers	Fluorochrome	Clone	Origin
**Immune cell types**	CD3	AF532	UCHT1	Invitrogen
	CD4	BV510	RPA-T4	BioLegend
	CD8	BV750	RPA-T8	BioLegend
	CD56	BV711	HCD56	BioLegend
	CD16	Ef450	eBioCB163	Invitrogen
	TCR-γδ	BV480	11F2	BD Biosciences
	CD19	BV750	HIB19	Biolegend
	CD14	AF647	MOP9	BD Biosciences
	CD123	PerCpCy5.5	7G3	BD Pharmingen
	CD11c	BV605	3.9	BioLegend
**Immune Activation / Maturation**	CD45RA	FITC	REA562	Miltenyi Biotech
	CCR7	BV421	G043H7	Biolegend
	CD28	APC-R700	CD28.2	BD Biosciences
	CD25	BV786	M-A251	BD Biosciences
	HLA-DR	APCCy7	1243	Biolegend
	CD26	PE	BA5b	Biolegend
	CD39	PeCy7	A1	BioLegend
	CD38	PerCPeF710	HB7	Invitrogen
**Immune checkpoint**	PD1	BV650	EH12.2H7	BioLegend
	CTLA-4	PeCy5	BNI3	BD Biosciences
	KIR2DL1	APC	REA284	Miltenyi Biotech
	KIR3DL1/DL2	APC	REA970	Miltenyi Biotech
	KIR2DL2/DL3	APC	DX27	Miltenyi Biotech
	KIR2DL5	APC	REA955	Miltenyi Biotech
**T-cell function**	Foxp3	PeCF	236A/E7	BD Biosciences
**Viability**	Zombie	NIR		Biolegend

**Extended data Table 5:** mAb list for CD8^+^ T cells panel.

	Markers	Fluorochrome	Clone	Origin
**Immune cell types**	CD3	AF532	UCHT1	Invitrogen
	CD4	BV510	RPA-T4	BioLegend
	CD8	BV570	RPA-T8	BioLegend
	CD56	APC-Cy7	HCD56	BioLegend
**Immune Activation / Maturation**	CD45RA	BV421	HI100	BioLegend
	CCR7	BV785	G043H7	BioLegend
	CD28	APC-R700	CD28 .2	BD Biosciences
	Hélios	PE-Dazzle594	22F6	BioLegend
**Immune checkpoint**	NKG2A	PE-Vio770	REA110	Miltenyi Biotech
	KIR2DL1	APC	REA284	Miltenyi Biotech
	KIR3DL1/DL2	APC	REA970	Miltenyi Biotech
	KIR2DL2/DL3	APC	DX27	Miltenyi Biotech
	KIR2DL4	APC	REA768	Miltenyi Biotech
	KIR2DL5	APC	REA955	Miltenyi Biotech
	NKG2C	VioBright	REA205	Miltenyi Biotech
	Nkp30	PE-Cy5	Z25	Beckman Coulter
	Nkp44	PE-Cy5	Z231	Beckman Coulter
	Nkp46	PE-Cy5	BAB281	Beckman Coulter
	PD1	BV650	EH12.2H7	BioLegend
**Viability**	zombie	NIR		BioLegend

**Extended data Table 6:** mAb list for cytotoxic CD8^+^ T cells panel

	Markers	Fluorochrome	Clone	Origin
**Immune cell types**	CD45	PerCP	HI30	BioLegend
	CD3	AF532	UCHT1	Invitrogen
	CD4	BV510	RPA-T4	BioLegend
	CD8	BV570	RPA-T8	BioLegend
	CD56	APC-Cy7	HCD56	BioLegend
**Immune Activation / Maturation**	CD45RA	BV421	HI100	BioLegend
	CCR7	BV785	G043H7	BioLegend
	CD28	APC-R700	CD28 .2	BD Biosciences
	Helios	PE-Dazzle594	22F6	BioLegend
	Nkp30	PE-Cy5	Z25	Beckman Coulter
	Nkp44	PE-Cy5	Z231	Beckman Coulter
	Nkp46	PE-Cy5	BAB281	Beckman Coulter
**Immune checkpoint**	NKG2A	PE-Vio770	REA110	Miltenyi Biotech
	KIR2DL1	APC	REA284	Miltenyi Biotech
	KIR3DL1/DL2	APC	REA970	Miltenyi Biotech
	KIR2DL2/DL3	APC	DX27	Miltenyi Biotech
	KIR2DL5	APC	REA955	Miltenyi Biotech
	NKG2C	VioBright	REA205	Miltenyi Biotech
	PD1	BV650	EH12.2H7	BioLegend
**Imune cell function**	GrzB/perf	PerCP-cy5.5	QA16A02/B-D48	Biolegend
**Viability**	zombie	NIR		BioLegend

**Extended data Fig. 2:**
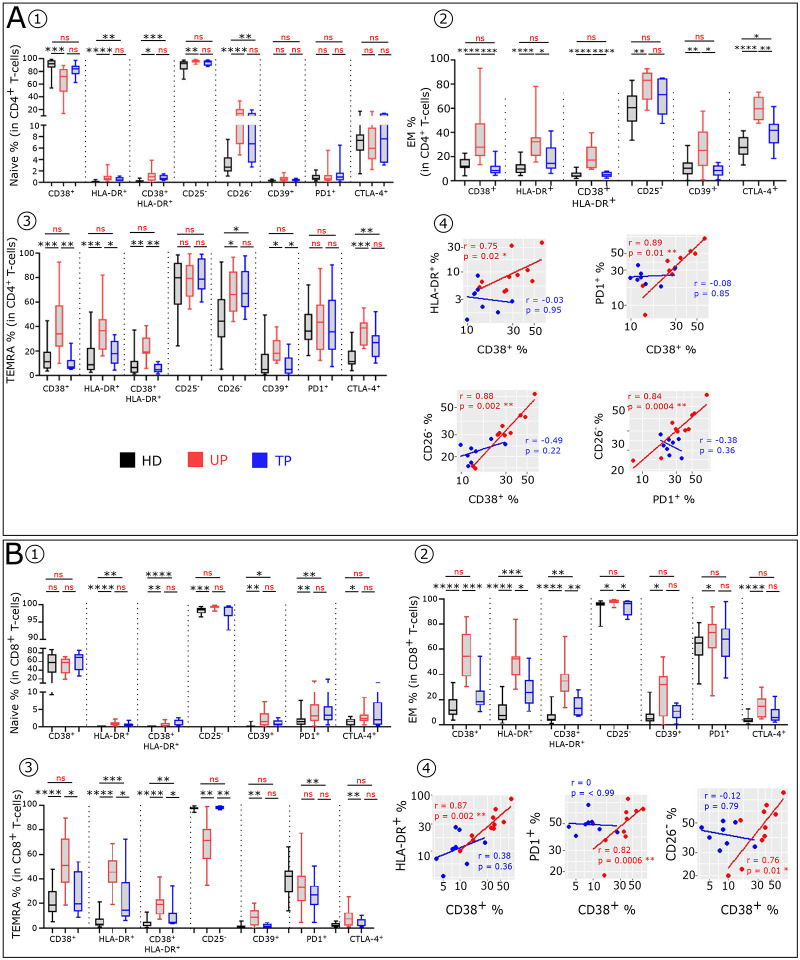
Comparative immune phenotypic analysis of CD4^+^ and CD8^+^ T-cell subsets in UP, TP and in HD. **(A)** Boxplots showing the expression of indicated marker in CD4^+^ naive **(A1)**, EM **(A2)** and TEMRA **(A3)** Tconv across the group (HD n=22, UP n=10 and TP n=8). **(A4)** Scatterplots showing relationships between the expression level of indicated markers in the CD4^+^CM subsets (UP n=10 and TP n=8). **(B)** Boxplots showing the expression of indicated marker in CD8^+^ naive **(B1)**, EM **(B2)** and TEMRA **(B3)** populations across the group (HD n=22, UP n=10 and TP n=8). **(B4)** Scatterplots showing relationships between the expression level of indicated markers in the CD8^+^CM subsets (UP n=10 and TP n=8).

**Extended data Fig. 3:**
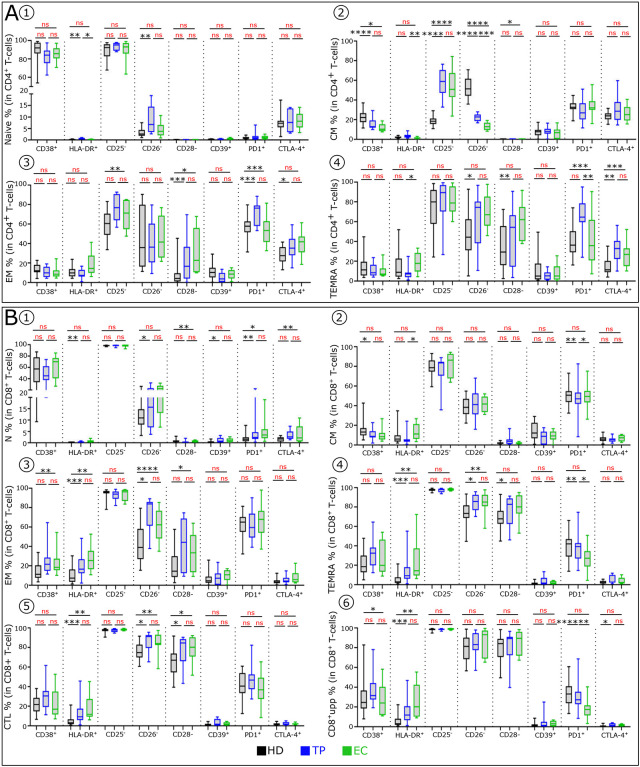
TP and EC share few blood immune cell anomalies. **(A)** Boxplots showing the expression of indicated marker in CD4^+^ naive **(A1)**, CM **(A2)**, EM **(A3)** and TEMRA **(A4)** populations across the group. (**B)** Boxplots showing the expression of indicated marker in CD8^+^ naive **(B1)**, CM **(B2)**, EM **(B3)** and TEMRA **(B4)** populations, and in TEMRA, CTL **(B5)** and CD8^+^supp **(B6)** across the group (HD n=22, EC n=10 and TP n=8).

## Abbreviations:

ADO: Adenosine
ADA: Adenosine Deaminase
cART: combined antiretroviral therapy
CD8 Tsupp: cytotoxic CD8^+^ suppressive T-cell
CM: Central Memory
CTL: CD8^+^ cytotoxic T lymphocyte
DC: Dendritic cells
EC: Elite Controllers
EM: Effector Memory
HD: Healthy Donors
IR: Immune Reaction
NK: Natural Killer
PBMCs: Peripheral Blood Mononuclear Cells
TP: Treated Patients
TEMRA: Terminal Differentiated Effector Memory
UP: Untreated Patients

## References

[1] Leon A. Rate and predictors of progression in elite and viremic HIV-1 controllers. AIDS 30, 1209–1220 (2016). 26854807 10.1097/QAD.0000000000001050

[2] Maggi P. Cardiovascular risk and dyslipidemia among persons living with HIV: a review. BMC Infect Dis 17, 551 (2017). 28793863 10.1186/s12879-017-2626-z PMC5550957

[3] Zhou Q. Malignancies in people living with HIV. AIDS Rev (2022) doi:10.24875/AIDSRev.21000057. 35319857

[4] Rasch M. G. Renal function and incidence of chronic kidney disease in HIV patients: a Danish cohort study. Scand J Infect Dis 44, 689–696 (2012). 22680981 10.3109/00365548.2012.673730

[5] Kruger M. J. & Nell T. A. Bone mineral density in people living with HIV: a narrative review of the literature. AIDS Res Ther 14, 35 (2017). 28747190 10.1186/s12981-017-0162-y PMC5530558

[6] Yuan N. Y. & Kaul M. Beneficial and Adverse Effects of cART Affect Neurocognitive Function in HIV-1 Infection: Balancing Viral Suppression against Neuronal Stress and Injury. J Neuroimmune Pharmacol 16, 90–112 (2021). 31385157 10.1007/s11481-019-09868-9 PMC7233291

[7] Finzi D. Identification of a reservoir for HIV-1 in patients on highly active antiretroviral therapy. Science 278, 1295–1300 (1997). 9360927 10.1126/science.278.5341.1295

[8] Wong J. K. Recovery of replication-competent HIV despite prolonged suppression of plasma viremia. Science 278, 1291–1295 (1997). 9360926 10.1126/science.278.5341.1291

[9] Neumann A. U. HIV-1 rebound during interruption of highly active antiretroviral therapy has no deleterious effect on reinitiated treatment. Comet Study Group. AIDS 13, 677–683 (1999). 10397562 10.1097/00002030-199904160-00008

[10] Davey R. T. HIV-1 and T cell dynamics after interruption of highly active antiretroviral therapy (HAART) in patients with a history of sustained viral suppression. Proc Natl Acad Sci U S A 96, 15109–15114 (1999). 10611346 10.1073/pnas.96.26.15109 PMC24781

[11] Deeks S. G. & Walker B. D. Human Immunodeficiency Virus Controllers: Mechanisms of Durable Virus Control in the Absence of Antiretroviral Therapy. Immunity 27, 406–416 (2007). 17892849 10.1016/j.immuni.2007.08.010

[12] Lambotte O. HIV Controllers: A Homogeneous Group of HIV-1--Infected Patients with Spontaneous Control of Viral Replication. Clinical Infectious Diseases 41, 1053–1056 (2005). 16142675 10.1086/433188

[13] Jiang C. Distinct viral reservoirs in individuals with spontaneous control of HIV-1. Nature 585, 261–267 (2020). 32848246 10.1038/s41586-020-2651-8 PMC7837306

[14] Le Buanec H .Early Elevated IFNα Identified as the Key Mediator of HIV Pathogenesis and its low level a allmark of Elite Controllers. Submitted.

[15] Cocchi F. Identification of RANTES, MIP-1 alpha, and MIP-1 beta as the major HIVsuppressive factors produced by CD8+ T cells. Science 270, 1811–1815 (1995). 8525373 10.1126/science.270.5243.1811

[16] Zagury D. C-C chemokines, pivotal in protection against HIV type 1 infection. Proc Natl Acad Sci U S A 95, 3857–3861 (1998). 9520457 10.1073/pnas.95.7.3857 PMC19927

[17] Zagury D. Interferon alpha and Tat involvement in the immunosuppression of uninfected T cells and C-C chemokine decline in AIDS. Proc Natl Acad Sci U S A 95, 3851–3856 (1998). 9520456 10.1073/pnas.95.7.3851 PMC19926

[18] Poli A. CD56 ^bright^ natural killer (NK) cells: an important NK cell subset. Immunology 126, 458–465 (2009). 19278419 10.1111/j.1365-2567.2008.03027.x PMC2673358

[19] Forconi C. S. A New Hope for CD56negCD16pos NK Cells as Unconventional Cytotoxic Mediators: An Adaptation to Chronic Diseases. Front Cell Infect Microbiol 10, 162 (2020). 32373555 10.3389/fcimb.2020.00162 PMC7186373

[20] Schiavon V. Microenvironment tailors nTreg structure and function. Proc Natl Acad Sci USA 116, 6298–6307 (2019). 30846549 10.1073/pnas.1812471116 PMC6442590

[21] Cha L., de Jong E., French M. A. & Fernandez S. IFN-α Exerts Opposing Effects on ActivationInduced and IL-7–Induced Proliferation of T Cells That May Impair Homeostatic Maintenance of CD4 ^+^ T Cell Numbers in Treated HIV Infection. J.I. 193, 2178–2186 (2014). 10.4049/jimmunol.1302536 25063872

[22] Deeks S. G. Immune activation set point during early HIV infection predicts subsequent CD4+ T-cell changes independent of viral load. Blood 104, 942–947 (2004). 15117761 10.1182/blood-2003-09-3333

[23] Mellors J. W. Prognosis in HIV-1 Infection Predicted by the Quantity of Virus in Plasma. Science 272, 1167–1170 (1996). 8638160 10.1126/science.272.5265.1167

[24] Zagury D. Long-term cultures of HTLV-III--infected T cells: a model of cytopathology of T-cell depletion in AIDS. Science 231, 850–853 (1986). 2418502 10.1126/science.2418502

[25] Orenstein J. M., Fox C. & Wahl S. M. Macrophages as a source of HIV during opportunistic infections. Science 276, 1857–1861 (1997). 9188531 10.1126/science.276.5320.1857

[26] Stacey A. R. Induction of a Striking Systemic Cytokine Cascade prior to Peak Viremia in Acute Human Immunodeficiency Virus Type 1 Infection, in Contrast to More Modest and Delayed Responses in Acute Hepatitis B and C Virus Infections. J Virol 83, 3719–3733 (2009). 19176632 10.1128/JVI.01844-08 PMC2663284

[27] Hardy G. A. D. Interferon-α is the primary plasma type-I IFN in HIV-1 infection and correlates with immune activation and disease markers. PLoS One 8, e56527 (2013). 23437155 10.1371/journal.pone.0056527 PMC3577907

[28] Cha L. Interferon-alpha, immune activation and immune dysfunction in treated HIV infection. Clin Transl Immunology 3, e10 (2014). 25505958 10.1038/cti.2014.1 PMC4232062

[29] Dondi E., Rogge L., Lutfalla G., Uzé G. & Pellegrini S. Down-modulation of responses to type I IFN upon T cell activation. J Immunol 170, 749–756 (2003). 12517937 10.4049/jimmunol.170.2.749

[30] Le Buanec H. IFN- and CD46 stimulation are associated with active lupus and skew natural T regulatory cell differentiation to type 1 regulatory T (Tr1) cells. Proceedings of the National Academy of Sciences 108, 18995–19000 (2011). 10.1073/pnas.1113301108 PMC3223453 22065791

[31] Dutrieux J. Modified interferon-α subtypes production and chemokine networks in the thymus during acute simian immunodeficiency virus infection, impact on thymopoiesis. AIDS 28, 1101–1113 (2014). 24614087 10.1097/QAD.0000000000000249

[32] Stoddart C. A., Keir M. E. & McCune J. M. IFN-alpha-induced upregulation of CCR5 leads to expanded HIV tropism in vivo. PLoS Pathog 6, e1000766 (2010). 20174557 10.1371/journal.ppat.1000766 PMC2824759

[33] Lazear H. M., Schoggins J. W. & Diamond M. S. Shared and Distinct Functions of Type I and Type III Interferons. Immunity 50, 907–923 (2019). 30995506 10.1016/j.immuni.2019.03.025 PMC6839410

[34] Migueles S. A. HLA B*5701 is highly associated with restriction of virus replication in a subgroup of HIV-infected long term nonprogressors. Proc Natl Acad Sci U S A 97, 2709–2714 (2000). 10694578 10.1073/pnas.050567397 PMC15994

[35] Claireaux M. Low CCR5 expression protects HIV-specific CD4+ T cells of elite controllers from viral entry. Nat Commun 13, 521 (2022). 35082297 10.1038/s41467-022-28130-0 PMC8792008

[36] Rolland M. Molecular dating and viral load growth rates suggested that the eclipse phase lasted about a week in HIV-1 infected adults in East Africa and Thailand. PLoS Pathog 16, e1008179 (2020). 32027734 10.1371/journal.ppat.1008179 PMC7004303

[37] Lian X. Signatures of immune selection in intact and defective proviruses distinguish HIV-1 elite controllers. Sci Transl Med 13, eabl4097 (2021). 34910552 10.1126/scitranslmed.abl4097 PMC9202005

[38] Salazar-Gonzalez J. F. Genetic identity, biological phenotype, and evolutionary pathways of transmitted/founder viruses in acute and early HIV-1 infection. J Exp Med 206, 1273–1289 (2009). 19487424 10.1084/jem.20090378 PMC2715054

[39] Van der Sluis R. M. Diverse effects of interferon alpha on the establishment and reversal of HIV latency. PLoS Pathog 16, e1008151 (2020). 32109259 10.1371/journal.ppat.1008151 PMC7065813

[40] Schwartz O., Maréchal V., Le Gall S., Lemonnier F. & Heard J. M. Endocytosis of major histocompatibility complex class I molecules is induced by the HIV-1 Nef protein. Nat Med 2, 338–342 (1996). 8612235 10.1038/nm0396-338

[41] Fisher A. G. The trans-activator gene of HTLV-III is essential for virus replication. Nature 320, 367–371 (1986). 3007995 10.1038/320367a0

[42] Herbeuval J.-P. Regulation of TNF-related apoptosis-inducing ligand on primary CD4+ T cells by HIV-1: role of type I IFN-producing plasmacytoid dendritic cells. Proc Natl Acad Sci U S A 102, 13974–13979 (2005). 16174727 10.1073/pnas.0505251102 PMC1224361

[43] Terawaki S. IFN-α directly promotes programmed cell death-1 transcription and limits the duration of T cell-mediated immunity. J Immunol 186, 2772–2779 (2011). 21263073 10.4049/jimmunol.1003208

